# Colored cotton wastes valuation through thermal and catalytic reforming of pyrolysis vapors (Py-GC/MS)

**DOI:** 10.1038/s41598-021-95043-1

**Published:** 2021-08-09

**Authors:** Janduir Egito da Silva, Guilherme Quintela Calixto, Rodolfo Luiz Bezerra de Araújo Medeiros, Marcus Antônio de Freitas Melo, Dulce Maria de Araújo Melo, Luiz Paulo de Carvalho, Renata Martins Braga

**Affiliations:** 1 Instituto Federal do Rio Grande do Norte, Campus Caicó, Caicó, RN Brazil; 2 Programa de Pós-Graduação em Engenharia Química, Universidade Federal do Rio Grande do Norte, Natal, RN Brazil; 3 Laboratório de Tecnologia Ambiental, Universidade Federal do Rio Grande do Norte, Natal, RN Brazil; 4 Departamento de Engenharia Química, Universidade Federal do Rio Grande do Norte – UFRN, Natal, RN Brazil; 5 Instituto de Química, Universidade Federal do Rio Grande do Norte, Natal, RN Brazil; 6 Empresa Brasileira de Pesquisa Agropecuária, Embrapa Algodão, Campina Grande, PB Brazil; 7 EAJ, Universidade Federal do Rio Grande do Norte, Macaíba, RN Brazil

**Keywords:** Environmental sciences, Energy science and technology

## Abstract

This study aims to analyze the products of the catalytic pyrolysis of naturally colored cotton residues, type BRS (seeds from Brazil), called BRS-Verde, BRS-Rubi, BRS-Topázio and BRS-Jade. The energy characterization of biomass was evaluated through ultimate and proximate analysis, higher heating value, cellulose, hemicellulose and lignin content, thermogravimetric analysis and apparent density. Analytical pyrolysis was performed at 500 °C in an analytical pyrolyzer from CDS Analytical connected to a gas chromatograph coupled to the mass spectrometer (GC/MS). The pyrolysis vapors were reformed at 300 and 500 °C through thermal and catalytic cracking with zeolites (ZSM-5 and HZSM-5). It has been noticed that pyrolysis vapor reforming at 500 °C promoted partial deoxygenation and cracking reactions, while the catalytic reforming showed better results for the product deoxygenation. The catalyst reforming of pyrolysis products, especially using HZSM-5 at 500 °C, promoted the formation of monoaromatics such as benzene, toluene, xylene and styrene, which are important precursors of polymers, solvents and biofuels. The main influence on the yields of these aromatic products is due to the catalytic activity of ZSM-5 favored by increased temperature that promotes cracking reactions due expanded zeolites channels.

## Introduction

Brazil is a major producer of cotton, accounting for approximately 11% of world production with 64,519.67 thousand tons, behind only countries such as the United States, China and India^1^. The cultivation of colored fiber cotton has stood out in recent years due to the sustainability of the production process of the fabric that does not require dyeing, reducing water consumption, cost, and treatment steps of the generated effluents^2^. The fabric made with naturally colored fibers, in addition to contributing to the environment, has been shown to be an alternative for people who are allergic to the dyes used in the coloring of white cotton fabrics. In addition, the elimination of the dyeing process can save up to one half of the cost of preparing textiles and also saves on disposal costs for toxic dye waste^3^.

The naturally colored cotton cultivars of Brazil have been developed by EMBRAPA (Empresa Brasileira de Pesquisa Agropecuária), vary between green, red and light brown colors and are called by Brazil of seeds (BRS) as BRS-Verde, BRS-Rubi, BRS-Topázio and BRS-Jade, and are low-cost produced consistently in semi-arid regions in northeast Brazil. The Brazilian agricultural production of this type of cotton generates tons of underutilized waste annually, approximately 2 times greater than the production of fibers^4^. These residues could be used for energy generation, but their main destination is direct landfill. However, this practice demands a high amount of energy and, in some cases, degrades the soil structure^4,5^.

The use of cultural residues from naturally colored cotton becomes interesting for the generation of distributed energy or chemical products, considering that different cultivars are being developed and that the parts (stalks and shell) have similar energy potentials, according to a reported study by Silva et al.^4^. Energy could be generated with agricultural waste from colored cotton (17 GJ ton^−1^), and a way to take advantage of this energy potential is through the pyrolysis process, especially catalytic flash pyrolysis for the production of biofuels and/or bioproducts of greater added-value.

Catalytic flash pyrolysis has been successfully used to deoxygenate bio-oil and improve its energy properties, in addition to increasing the yield of aromatic compounds, such as benzene, toluene, ethylbenzene and xylene (BTEX)^6^, which are high-valued compounds obtained mainly from the oil industry. The use of zeolites ZSM-5 and HZSM-5 in the catalytic flash pyrolysis process has been consolidating, with emphasis on HZSM-5, which has shown good efficiency in the production of deoxygenated hydrocarbons^7^. The high specific area and acidity of these catalysts are indispensable to promote the deoxygenation reactions of the pyrolysis products leading to renewable chemicals of industrial interest. Hu et al.^8^ studied the best pore size for deoxygenation of biomass pyrolysis products, showing the HZSM-5 effectivity in deoxygenating the products. Liu et al.^9^ also showed the efficiency in promoting monocyclic aromatics when using HZSM-5 and modified HZSM-5 catalysts. Barbosa et al.^10^ confirmed the high deoxygenation performance of a low-cost synthesized HZSM-5 at the catalytic pyrolysis of pineapple crown leaves, with high production of BTEX compounds.

Therefore, the objective of this work is to evaluate the production of renewable aromatic compounds through the flash pyrolysis of naturally colored cotton waste through thermal and catalytic cracking of pyrolysis vapors, using ZSM-5 and HZSM-5 at 300 and 500 °C, and also to evaluate the activity of these catalysts. The valorization of naturally colored cotton wastes is presented in this study as an alternative for the production of energy, biofuels and value-added bioproducts. In this study it is presented the energetic potential of the biomass, flash pyrolysis and its upgrading through two routes: thermic and catalytic reforming.

## Materials and methods

### Biomass characterization

**Figure 1:**
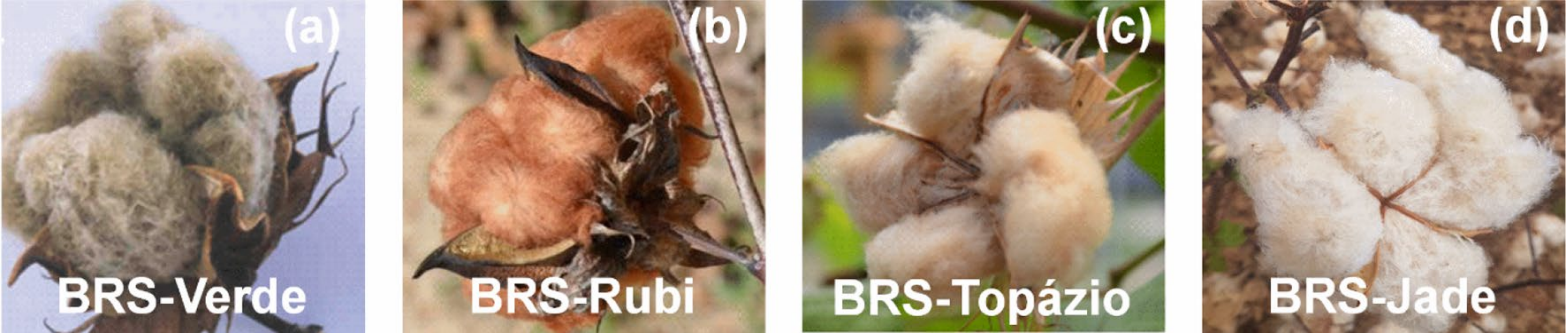
Cultivars BRS-Verde (**a**), BRS-Rubi (**b**), BRS-Topázio (**c**), BRS-Jade (**d**).

The cultural remains (stalk and shells) of the cultivars BRS-Brazil seeds called BRS-Verde (V), BRS-Rubi (S), BRS-Topázio (T) and BRS-Jade (J) (Fig. 1), were collected on a farm belonging to the company of agricultural research of Rio Grande do Norte called EMPARN, located at Apodi, RN.

The biomass used in this study resulted from the mixing between stalks and shell in equal proportions, from the different naturally colored cotton cultivars, after sun-dried for 16 h for the stabilization of moisture level, ground in a knife mill and sieved to obtain particles ranging in size between 0.104 and 0.074 mm. The energy characteristics of the biomass were evaluated through the apparent density analysis, determined in accordance with American Society for Testing and Materials (ASTM E 873-82), proximate analysis, which consists of the determination of the moisture content (ASTM E 871-82), volatile (ASTM E 872 and E 897), ash (ASTM E 1755-01) and fixed carbon by difference. The higher heating value (HHV) has been measured using a bomb calorimeter following the ASTM E711-87 standard. The constituent metals of the biomass ash were determined by X-ray fluorescence (XRF) by dispersive energy, using a Shimadzu EDX-820 equipment. X-ray fluorescence spectra were obtained using about 300 mg of biomass in powder form.

The ultimate chemical analysis was performed on the 2400 Series II elemental analyzer (CHNS/O) from PerkinElmer. The evaluation of the thermal behavior of the biomass samples was carried out on SDT Q600 thermogravimetric analyzer from TA Instruments. The analysis was carried out at room temperature at 900 °C, with a heating rate of 10 °C min^−1^, under a flow of 100 mL min^−1^ of N_2_, using approximately 10 mg of the biomass.

The cellulose and hemicellulose content were determined through the methodology developed by Van Soest and Wine (1967), using the Acid Detergent Fiber (ADF) and Neutral Detergent Fiber (NDF) tests; the lignin content was determined directly by the Klason method^11^.

### Catalyst

The commercial ZSM-5 used was obtained from PQ Corporation, with defined characteristics, cited by Corma et al.^12^, which proceeded to the ion exchange process to obtain HZSM-5. The synthesis of HZSM-5 occurred according to Barbosa et al.^10^, using 5 g of ZSM-5 and ammonium chloride (NH_4_Cl) solution (1 M) for the ion exchange. The obtained solid was dried in an oven at 100 °C. The crystalline structure of the ZSM-5 and HZSM-5 catalysts was analyzed by X-ray diffraction (XRD) using CuKα radiation (λ = 1.5409 Å) operating at 2° min^−1^ with voltage and amperage of 40 kV and 30 mA, respectively, in a (XRD 7000) Shimadzu diffractometer. The Rietveld refinement was performed in the Maud program (version 2.2) using the crystallographic data corresponding to the files identified in the diffractograms. The characteristic bands of the main connections between Al and Si were identified through spectroscopy in the infrared region (FT-IR), using the Shimadzu equipment, model IR Prestige-21. The chemical composition was determined by X-ray fluorescence (XRF), using a Shimadzu EDX-820 equipment.

### Pyrolysis

Biomass analytical (flash) pyrolysis took place in a 5200 HP-R micro pyrolyzer from CDS Analytical connected to a gas chromatograph coupled to mass spectrometry detection (GC/MS) 3900 from VARIAN. Approximately 1 mg of the biomass was trapped in a small quartz tube by inserting glass wool at its ends, and heated to 500 °C, with a heating rate of 10 °C ms^−1^, by a platinum filament that surrounds the quartz tube. The vapors from the thermal decomposition of the biomass were dragged with 50 mL min^−1^ of N_2_, being subsequently stored in a Tenax trap and desorbed at 300 °C, for 4 min. The pyrolysis vapors, stored in the trap, were injected, through the 1/50 split mode, into the GC–MS using a VF-5MS chromatographic column (30 m × 0.25 mm × 0.25um). The heating temperature programming of the column was adjusted to start the analysis at 40 °C, holding at this temperature for 2 min, then heated at 10 °C min^−1^ to 280 °C, holding at this temperature for 14.5 min. The semi-quantitative approach was based on the internal normalization method, considering the area/mass (Area/mg) of the peaks identified in the chromatogram by searching National Institute Standards and Technology (NIST) mass spectra library when similarity above 85%.

### Reforming of pyrolysis vapors

After pyrolysis at 500 °C, the produced vapors were directed to a catalytic bed with controlled temperature through a 6 vias valve heated at 300 °C. The thermal upgrading of pyrolysis vapors products was developed at 300 and 500 °C into the catalytic bed using only quartz wool. The temperatures of 300 and 500 °C were chosen based on the highest decomposition rate temperature and the end of most mass loss stages, respectively. The products were transported to the GC-MS through a transfer line heated to 300 °C. The GC-MS products separation and analysis carried out using the experimental analytical conditions described in item 2.3.

Catalytic reforming of biomass was performed directing the pyrolysis vapors to the catalytic fixed bed containing 10 mg of catalysts ZSM-5 and HZSM-5 heated at 300 and 500 °C, through the opening of a valve that connects the catalyst with the pyrolysis system. The catalytic reforming products were transported through a transfer line heated to 300 °C and injected in the GC-MS with split mode 1/50 following the experimental analytical conditions described in item 2.3.

## Results and discussion

### Biomass characterization

The biomass showed characteristics that make it suitable for the production of bio-oil through the flash pyrolysis process, such as high HHV (17 MJ kg^−1^), higher than common biomasses, such as sugarcane bagasse (Santos et al., 2020)^13^, ash content less than 10% and volatile content similar to switchgrass^14^ (Table 1). The heating value found was similar to other lignocellulosic biomasses, such as rice husks (17.95 MJ kg^−1^), peanut husks (20.61 MJ kg^−1^) and wheat straw (17.36 MJ kg^−1^)^15^.

**Table 1:** Colored cotton waste biomass characterization.

Analysis	Biomass
Moisture (%)	9.3 ± 0.06
Volatile (%)	69.0 ± 0.03
Ash (%)	8.2 ± 0.04
Fixed Carbon^a^ (%)	13.5 ± 0.04
Higher heating value (MJ kg^−1^)	17.0 ± 0.05
Bulk density (kg m^−3^)	350 ± 0.04
Carbon (%)	41.1 ± 0.07
Hydrogen (%)	6.4 ± 0.05
Nitrogen (%)	1.62 ± 0.04
Oxygen^b^ (%)	51.0 ± 0.03
Cellulose (%)	38.2 ± 0.04
Hemicellulose (%)	25.8 ± 0.05
Lignin (%)	14.7 ± 0.02

^a^Determined by difference, fixed carbon% = 100% − moisture% − volatile% − ash%.

^b^Determined by difference, O% = 100% − carbon% − hydrogen% − nitrogen%.

Zhang et al. showed that the cotton stalk from white cotton presented different energetic characteristics when compared to natural coloured one in this study, with 79.87% volatile content, 3.75% ash content, 46.34% carbon content, 43.18% oxygen content and 28.5% lignin, indicating that its bio-oil could have substantially different properties.

From Table 1, it can be seen that the O/C ratio of 1.24 and H/C of 0.16 indicate oxygen content higher than 50%, which can results in a bio-oil with many oxygenated compounds and, therefore, chemically unstable and with low calorific value, requiring the use of catalytic upgrading to improve its quality.

The ash content (8.2%) corresponds to the minerals absorbed during the growth of the plant that form the inorganic part of the biomass after burning; however, some of the minerals present in the ash are particulate impurities that come into contact with the biomass during the harvesting^17^. The ash chemical composition was 53.4% potassium (K), 32.5% calcium (Ca), 5.6% sulfur (S), 2.6% iron (Fe), 1.6% magnesium (Mg), 1.6% silicon (Si) and traces of other elements such as phosphorus (P). The minerals present in the biomass ash act as a catalyst in the pyrolysis process^18^, and can affect the yield and products of pyrolysis, reducing the yield of bio-oil and increasing the production of coal and gas, through depolymerization reactions that form oxygenated molecules of lower molecular weight^19^. The presence of K in biomass ash has a catalytic effect on pyrolysis, favoring the yield of phenolics in bio-oil^20^. The alkali and alkaline earth metals naturally present in biomass ash serve as strong ring-fragmentation catalysts, likely due to ion–dipole forces, yielding to light acid oxygenated compounds and a chemical unstable bio-oil^21^.

**Figure 2:**
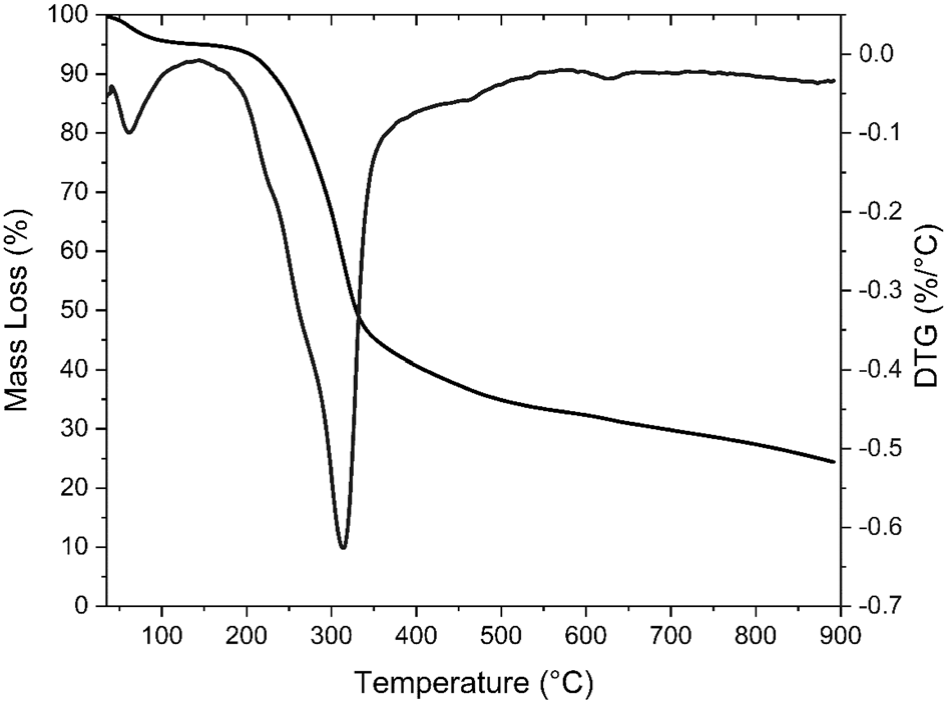
Biomass thermogravimetric curves (TG/DTG).

The thermogravimetric analysis (Fig. 2) shows that the first stage of biomass decomposition occurred between 35 and 150 °C, referring to a mass loss of approximately 5% of moisture.

The remaining steps correspond to the decomposition of the polymeric structures present in the biomass, such as hemicellulose, cellulose and lignin. The decomposition of the polysaccharides that make up hemicellulose occurs at a moderate reaction intensity, followed by cellulose degradation, which presents a well-defined peak because it is a homogeneous polysaccharide formed by glucose units, with maximum decomposition temperature at 310 °C, observed in the differential thermogravimetric curve (DTG). Lignin decomposes over a wide temperature range, which largely overlaps the mass loss events related to hemicellulose and cellulose^22^. The decomposition of hemicellulose and cellulose is responsible for most of the condensable volatiles that originate the bio-oil, which are mainly generated in the range of 200 to 500 °C^23^. In this stage, there is a 64.1% mass loss. This result corroborates the volatile content shown in Table 1.

### Catalyst

**Figure 3:**
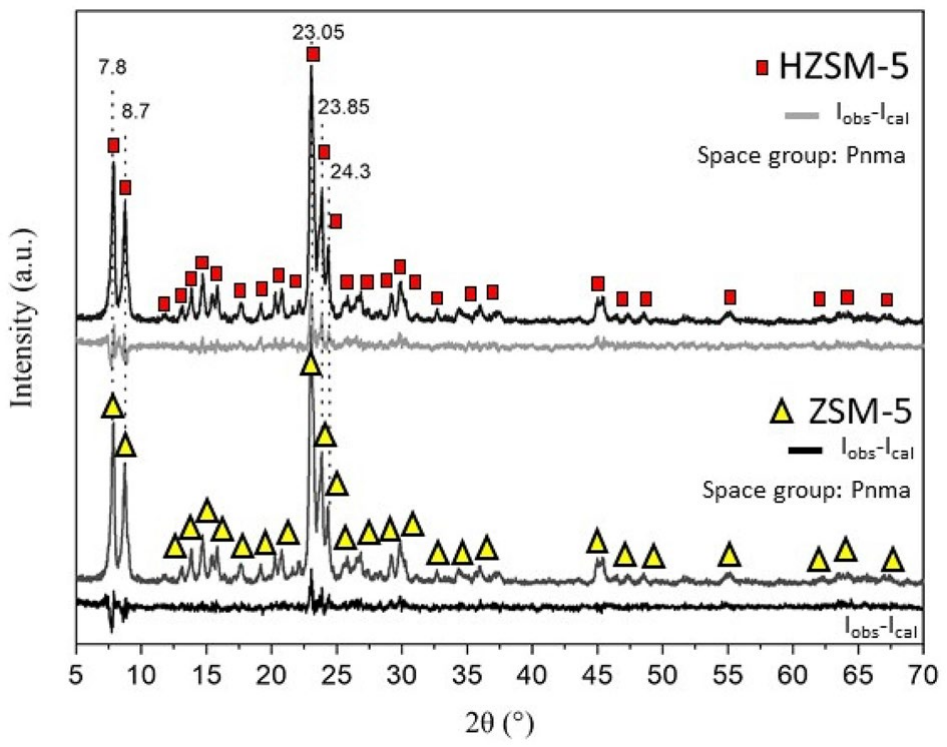
XRD pattern of ZSM-5 and HZSM-5.

The crystalline structures characteristic of the zeolites ZSM-5 and HZSM-5 were confirmed through X-ray diffraction results (Fig. 3), according to crystallographic records: 01-079-1638 and 01-080-0922 (JCPDS files), respectively, where the main diffraction lines located at positions 2θ = 7.8°, 8.7°, 23.05°, 23.85°, and 24.3° are characteristic of the structure of these zeolites.

The crystalline structure of the orthorhombic type with the Pnma space group was confirmed by the Rietveld method, where the network parameters presented in Table 2 are characteristic of zeolite materials. A slight increase in the dimensions of the unit cell was observed for the zeolite subjected to the ion exchange treatment (HZSM-5), and no secondary phase was identified.

**Table 2:** Rietveld refinement data of the catalyst.

Rietveld refinement data	Catalysts	
	ZSM-5	HZSM-5
Space group	Pnma	Pnma
λ (Å), Cu-kα	1.5409	1.5409
Data acquisition temperature T (°C)	25	25
a (Å)^a^	20.0605	20.1447
b (Å)^a^	19.8193	19.9357
c (Å)^a^	13.3404	13.4110
V (Å^3^)^b^	5303.9	5385.8
GoF^c^	1.97	1.88
Rw (%)	18.35	17.12
CIF used^d^	66,648	68,734

^a^Cell parameter.

^b^Cell volume.

^c^Goodness of fit.

^d^Crystallograph information.

The Si/Al ratio of the zeolites was confirmed by XRF, in which 84.50% SiO_2_ and 15.18% Al_2_O_3_ are observed for ZSM-5 and 84.57% SiO_2_ and 15.12% Al_2_O_3_ for HZSM-5, which confirms that the acidification step did not change the Si/Al ratio of 18.

**Figure 4:**
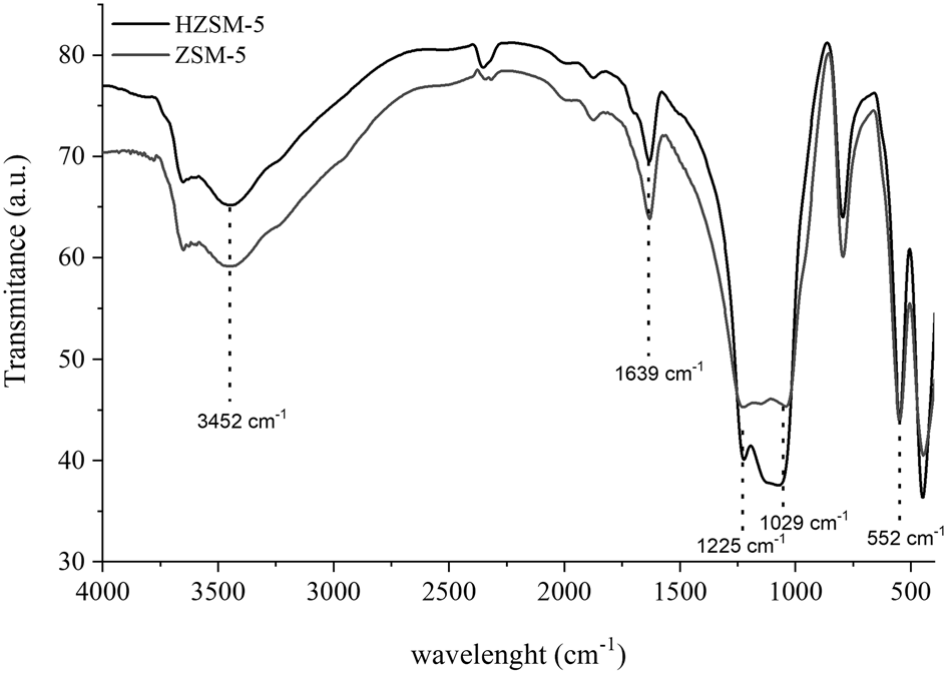
FT-IR spectra of ZSM-5 and HZSM-5.

Figure 4 shows the absorption spectra of the infrared region of the ZSM-5 and HZSM-5 zeolites. A broad band of approximately 3452 cm^−1^ is observed, which represents the axial deformation of O–H of the Si–OH group, commonly observed between 3700 and 3200 cm^−1^ ^24^. At 1639 cm^−1^, the deformation band for the water molecule external to the zeolite structure is verified. Asymmetric internal and external stretches occur in 1225 and 1029 cm^−1^, respectively, representing the Si–O–Si siloxane groups^25^. In this region, a higher intensity stretch is observed for HZSM-5, which is due to the expansion of the unit cell, previously shown in Table 2. The absorption bands at 552 and 445 cm^−1^ represent the vibration of double rings of five members of the three-dimensional porous structure of both ZSM-5 and HZSM-5 zeolites^26^.

### Pyrolysis

The pyrolysis of biomass at 500 °C resulted in several oxygenated compounds from different organic groups (Table 3), such as aldehydes, ketones, phenolics, furans, ethers and light oxygenated compounds containing between 1 and 4 carbon atoms (C_1_-C_4_), characteristic of the decomposition of lignocellulosic materials^15,23,27^.

**Table 3:** Conventional pyrolysis products distribution at 500 °C.

RT^a^ (min)	Compound (formula)	Group	Area/mg (10^5^)
1.56–4.11	C_1_–C_4_	Organic acids	∑ = 77.6
4.70	2,5-Dimethyl-furan (C_6_H_8_O)	Furan	1.7
5.30	1-Methyl-1H-pyrrole (C_5_H_7_N)	Nitrogenated	4.5
5.60	Pyrrole (C_4_H_5_N)	Nitrogenated	1.6
5.88	Toluene (C_7_H_8_)	Aromatic	6.2
6.34	Cyclopentanone(C_5_H_8_O)	Ketone	1.7
7.25	3-Furaldehyde (C_5_H_4_O_2_)	Aldehyde	10.2
8.11	p-Xylene (C_8_H_10_)	Aromatic	2.8
8.89	2-Methyl-cyclopenten-1-one (C_6_H_8_O)	Ketone	5.0
8.90	2-Ethyl-5-methylfuran (C_7_H_10_O)	Furan	5.0
10.39	Phenol (C_6_H_6_O)	Phenol	4.5
11.78	2,3-Dimethyl-cyclopenten-1-one (C_7_H_10_O)	Ketone	2.6
11.98	o-Methyl-phenol (C_7_H_8_O)	Phenol	2.3
12.38	p-Methyl-phenol (C_7_H_8_O)	Phenol	2.4
12.83	o-Methoxy-phenol (C_7_H_8_O_2_)	Ether/phenol	2.6
13.10	4,4-Dimethyl-cyclohexen-1-one (C_8_H_2_O)	Ketone	1.5
13.89	2,3-Dimethylphenol (C_8_H_10_O)	Phenol	1.2
14.60	2-Methoxy-5-methylphenol (C_8_H_10_O_2_)	Ether/phenol	0.5
14.86	o-Methoxy-m-methylphenol (C_8_H_10_O_2_)	Ether/phenol	0.6

^a^Retention time (min)

The high proportion of C_1_–C_4_ compounds is noteworthy among the pyrolysis products of this biomass, which are products from the cracking of larger molecules such as polysaccharides and lignin. These compounds (C_1_–C_4_) are characterized by different organic acids, such as acetic acid, acetic anhydride, methyl acetate, formic acid, and others that give bio-oil chemical instability and low energetic potential^28^.

The second highest yield was observed for phenolics, mainly phenol, methyl phenol and methoxylated phenols. The formation of phenols is mainly associated with the decomposition of lignin, which has a structure formed by phenylpropane, with o-methoxy-m-methylphenol being a primary product of the decomposition of coniferyl alcohol (G). The inorganics present in the ashes of the lignocellulosic biomass also contribute to the phenolics formation due to its catalytic effect that favors the decomposition reactions of the methoxy groups in phenols, changing the yield of the products^29^. The high number of phenolic compounds in the pyrolysis products of this biomass can be partly explained by the catalytic effect of the high K content (53.4%) on biomass ash^20^.

The presence of aldehydes, followed by ketones, especially of the cyclopentanone type, has also been identified, which are derived from the decomposition of hemicellulose and cellulose, and their presence may also have been corroborated by the high calcium content (32%) contained in the biomass ash. According to Case et al.^30^, calcium oxide acts in the cracking of levoglucosan and favors the formation of alkylated phenols, polyaromatic hydrocarbons and alkyl-substituted cyclopentenones. The presented results shows consistent differences when compared to the results obtained by Zhang et al*.*^16^ for the white cotton stalks, which composition was mainly phenols (41.4%), organic acids (8.6%), ketones (3.6%) and aldehydes (2.5%).

The main products resulting from flash pyrolysis followed by thermal cracking of ex-situ vapors (upgrading)—reforming of pyrolysis vapors in a fixed heated bed at 300 and 500 °C in the absence of the catalyst—are described in Table 4.

**Table 4:** Thermal pyrolysis products for 300 and 500 °C.

RT^a^ (min)	Compound (Formula)	Group	Area/mg (10^5^) Py300	Area/mg (10^5^) Py500
1.55–3.96	C_1_–C_4_	Organic acids	∑ = 306.1	∑ = 187.1
4.69	2,5-Dimethyl-furan (C_6_H_8_O)	Furan	7.4	3.1
5.86	Pyrrole (C_4_H_5_N)	Nitrogenated	26.5	2.5
5.90	Toluene (C_7_H_8_)	Aromatic	NI^b^	10.7
7.27	Furaldehyde (C_5_H_4_O_2_)	Aldehyde	26.2	24.5
7.95	Ethylbenzene (C_8_H_10_)	Aromatic	11.3	2.9
8.12	p-Xylene (C_8_H_10_)	Aromatic	6.4	6.5
8.32	Phenol (C_6_H_6_O)	Phenol	NI	4.4
8.59	Styrene (C_8_H_8_)	Aromatic	NI	2.0
8.69	o-Xylene (C_8_H_10_)	Aromatic	NI	1.0
8.90	2-Methyl-cyclopenten-1-one (C_6_H_8_O)	Ketone	8.2	9.4
10.42	Phenol (C_6_H_6_O)	Phenol	12.3	20.6
10.95	Benzofuran (C_8_H_6_O)	Aromatic/furan	NI	3.5
12.00	o-Methyl-phenol (C_7_H_8_O)	Phenol	6.0	6.1
12.40	m-Methyl-phenol (C_7_H_8_O)	Phenol	11.7	10.3
12.84	Mequinol (C_7_H8O_2_)	Phenol	25.6	NI
16.47	p-Ethyl-m-methoxy-phenol (C_9_H_12_O_2_)	Ether/phenol	6.3	NI
17.72	3,4-Dimethoxy-phenol (C_8_H_10_O_3_)	Ether/phenol	18.4	NI
19.4	o-Methoxy-p-(1-propenyl)-phenol (C_10_H_12_O_2_)	Ether/phenol	16.0	NI

^a^Retention time (min).

^b^Not identified.

The major compounds among pyrolysis products after ex-situ thermal reforming are light oxygenates C_1_-C_4_, that decrease their content with the temperature increase which shows the favoring of gas formation. It is also observed (Table 4) that the yield of aromatic compounds was higher at 500 °C, formed through the scission of the methoxylated phenol molecules, such as 3,4-dimethoxy-phenol, o-methoxy-p-(1-propenyl)-phenol and p-ethyl-m-methoxy-phenol, since C-O bonds require less energy (358 kJ mol^−1^) than C=C bonds (614 kJ mol^–1^) to be broken^4^. Thus, C–O bonds undergo fission at 500 °C resulting in monoaromatics such as toluene, styrene and xylene, and phenol that require temperatures greater than 500 °C to break their bonds. In general, it is observed that the increase in temperature promotes the formation of ketones, aromatics (monosubstituted and disubstituted) and phenol (common and monosubstituted), while decreasing the yield of disubstituted phenols and furans. The effect of cracking temperature can also be seen with the decrease in pyrrole yield, which changes from 26.5 × 10^5^ to 2.5 × 10^5^ Area mg^−1^ at temperatures of 300 to 500 °C, respectively, and occurs due to the splitting of CN bonds leading to light nitrogenous compounds such as NO_x_ and NH_3_ that are not detected by GC–MS. The results of the reforming of the pyrolysis vapors prove that the increase in temperature influences the yield of compounds that have greater thermal stability, but has not been sufficient for the deoxygenation of the pyrolysis products.

The main products of catalytic pyrolysis are described in Table 5, in which it is possible to observe that the results varied depending on the temperature and the type of catalyst used. In ascending order ZSM-5 and HZSM-5 promoted the formation of renewable monoaromatic compounds predominantly.

**Table 5:** Catalytic pyrolysis with ZSM-5 and HZSM-5 at 300 and 500 °C products distribution.

RT^a^ (min)	Compound	Group	ZSM-5_Py300 area/mg (10^5^)	HZSM-5_Py300 area/mg (10^5^)	ZSM-5_Py500 area/mg (10^5^)	HZSM-5-Py_500 area/mg (10^5^)
1.51–3.10	C1–C4	Organic acids	∑ = 2.2	∑ = 2.6	∑ = 24.7	∑ = 7.6
3.96	Benzene	Aromatic	1.6	1.8	5.7	27.0
5.87	Toluene	Aromatic	7.4	5.2	18.7	91.0
7.93	Ethylbenzene	Aromatic	1.1	1.1	4.2	6.5
8.10	1,3-Dimethylbenzene	Aromatic	5.8	9,0	24.2	59.0
8.64	p-Xylene	Aromatic	1.9	2.3	6.7	10.6
9.98	Propylbenzene	Aromatic	0.2	NI^b^	0.6	NI
10.15	2-Ethyl-1-methyl-benzene	Aromatic	2.5	3.1	11.6	3.3
10.42	Phenol	Phenol	NI	NI	1.1	NI
10.85	1,2,3-Trimethyl-benzene	Aromatic	2.4	4.8	2.1	9.5
10.94	Benzofuran	Furan	0,2	0,1	1,6	1,5
11.78	2,3-Dimethyl-cyclopenten-1-one	Ketone	1.0	1.0	2.1	4.0
12.00	o-Methyl-phenol	Phenol	0.5	NI	1.0	3.1
12.40	p-Methyl-phenol	Phenol	NI	NI	1.0	NI
12.73	2-Butenyl-benzene	Aromatic	1.4	1.0	4.2	NI
13.91	2-Methyl-1-propenyl-benzene	Aromatic	1.0	NI	2.6	1.6
14.74	3,4-Dihydro-2,2-dimethyl-1H-indane	Aromatic	0.4	0.4	1.9	NI
14.85	Naphtalene	Aromatic	0.5	0.6	2.0	10.2
16.9	Methylnaphthalene	Aromatic	0.5	0.5	2.5	9.2

According to Table 5, it is possible to state that both catalysts promoted the formation of benzene and toluene, especially at 500 °C, with emphasis on HZSM-5. The ZSM-5 catalysts significantly favor the formation of benzene, alkylbenzenes and polyaromatics through the aromatization reaction, since these types of catalysts tend to have desirable physicochemical properties such as strong Brønsted acidity, high water tolerance, and high thermal stability, necessary conditions for the production of aromatic hydrocarbons^31^.

^a^Retention time (min).

^b^Not identified.

**Figure 5:**
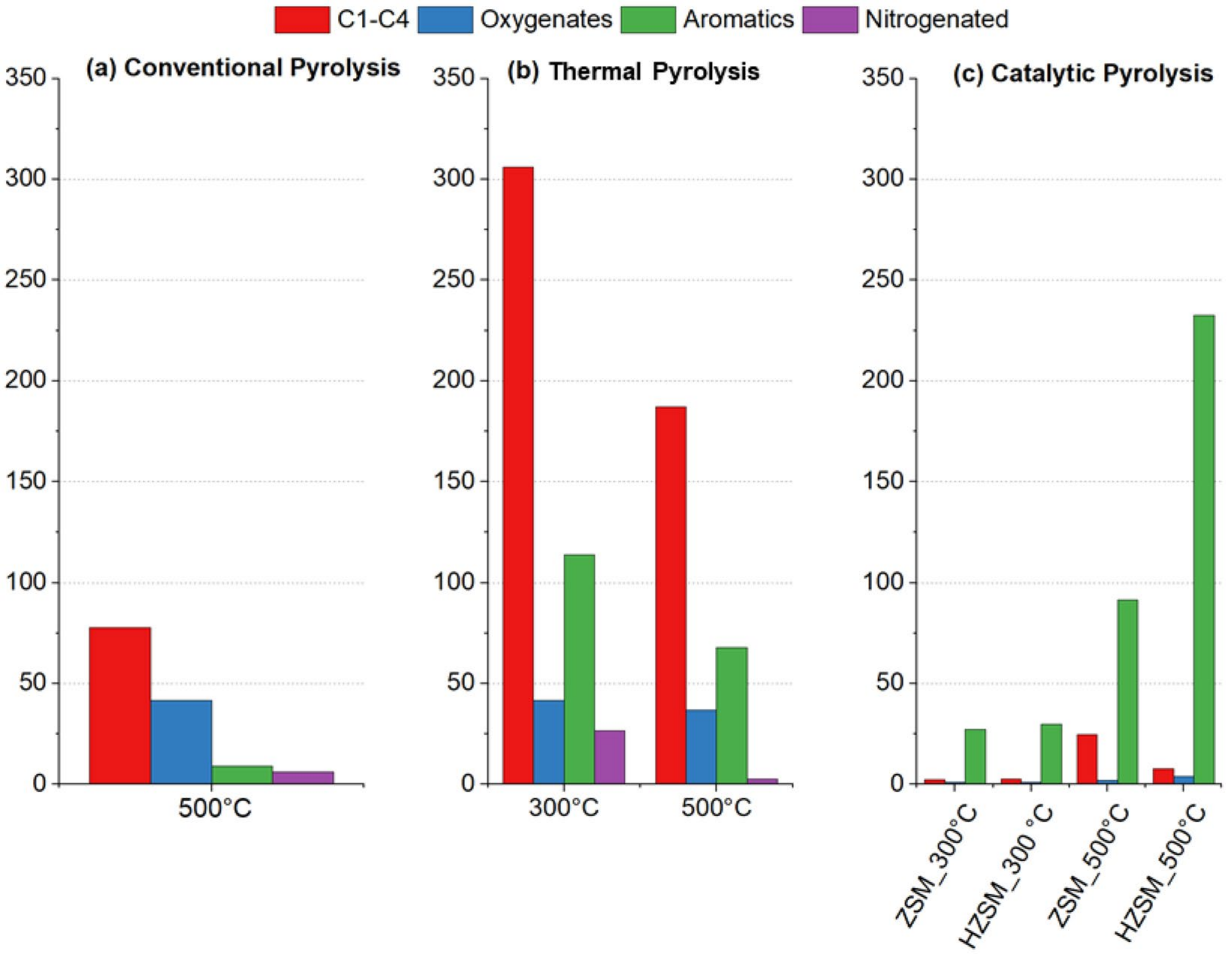
Pyrolysis products by groups.

According to Engtrakulet al.^32^, the acidic sites of aluminosilicate zeolites tend to be reasonably uniform in strength and located in well-defined pore structures, resulting in performance and stability in relation to amorphous acid catalysts. The ZSM-5 and HZSM-5 at 300 °C were unfavorable to the formation of ketones; however, at 500 °C, there is an increase in the amount of 2,3-dimethyl-cyclopenten-1-one, especially when the HZSM-5 was used, indicating that the increase in temperature can optimize the action of the acidic sites of this catalyst towards the formation of this compound, according to a similar study carried out by Iliopoulou et al.^33^. The pyrolysis products distribution from previous analysis is presented on Fig. 5.

The sum of condensable products from catalytic pyrolysis with ZSM-5 and HZSM-5 at 300 °C was much lower than at 500 °C. The same is applied for the thermal reforming: although it seems that the thermal pyrolysis produced a considerable amount of aromatics, the products of catalytic pyrolysis are much more concentrated. Thus, the catalytic bed at 300 °C is not the ideal condition for obtaining bio-oil with a high renewable aromatic content. It is also noticed that at 500 °C both catalysts, with emphasis on HZSM-5, promoted the formation of many condensable compounds, mainly BTX-type monoaromatics (benzene, toluene and xylene), in addition to polyaromatic compounds, such as naphthalene, from which, many can be separated from bio-oil by fractional distillation and, therefore, there may be the formation of a class of chemical compounds of commercial interest. HZSM-5 is more acidic than ZSM-5 and, combined with the expansion of the unit cell, observed from the DRX and FTIR (Figs. 3 and 4), promoted a greater formation of aromatic compounds due to the cracking of molecules with higher molecular masses, confirmed in a study by Shafaghat et al.^34^.

It can be observed (Fig. 5) that the yield of the products at both thermal upgrading temperatures (300 and 500 °C) was higher than the catalytic upgrading. The reactor temperature at 300 °C produced more oxygenates and nitrogenated compounds. At a temperature of 500 °C, it was observed that there was cracking and a slight aromatization, as well as a marked reduction in the yield of bio-oil products, caused by the consequent formation of gases such as NOx, CH_4_, CO and CO_2_, being the last two results from deoxygenation and decarbonylation reactions promoted in the catalytic bed. Thus, it can be said that the thermal upgrading (ex-situ) of the pyrolysis products at these temperatures is not enough to deoxygenate the bio-oil or to form satisfactory content of aromatics.

The results show that the thermal upgrading of pyrolysis vapors favored the formation of renewable aromatics from secondary reactions that led to deoxygenation of the primary pyrolysis products; however, the thermo-catalytic reforming of pyrolysis vapors has been more efficient, especially at higher temperature (500 °C), since the increase in temperature influences the catalyst structure for selectivity of these compounds^10^.

Condensable vapors formed by acids, alcohols, ketones, furans, esters and phenols on contact with the catalytic bed (ZSM-5 and HZSM-5) at different temperatures are cracked and undergo deoxygenation reactions, resulting in olefins (C_1_ to C_6_), which are aromatized to form benzenes and other aromatic compounds.

The catalytic pyrolysis is an alternative method to value colored cotton waste and obtain renewable chemicals, especially benzene, toluene and xylene. BTEX compounds are highly valued because of their large use as solvent and chemical precursors for resins, dyes, pesticides, having considerable different boiling points that can be easier separated from the bio-oil by using distillation methods. It is also important to note that the main source of these chemicals is the oil industry, highlighting the importance of the catalytic flash pyrolysis as a process to obtain green chemicals with zero emission.

Therefore, this study brings the possibility of the sustainable production of chemicals of industrial interest, such as BTXs, which can be obtained through the catalytic cracking of the natural coloured cotton wastes. The large-scale implementation of this project could boost the social and economic development due to income generation through the use and transformation of these biomass residues, also helping in the formation of the environmental awareness of society and conscious consumption without harming the environment. However, one of the main challenges is the lack of adequate investments, aimed at the development of small and medium-sized plants that can generate such products in a distributed manner.

## Conclusion

Colored cotton wastes are promising lignocellulosic biomass for the production of renewable aromatics through catalytic reforming of flash pyrolysis vapors, as they have adequate energy characteristics for thermochemical conversion, such as high energetic density. The main products of flash pyrolysis were light oxygenated compounds of carbon chains of up to 4 carbons such as methane and acetic acid, followed by phenols, aldehydes and ketones, respectively. Thermal improvement, especially at 500 °C, favored the yield of aromatic compounds, formed by the splitting of methoxylated phenol molecules, such as 3,4-dimethoxyphenol, o-methoxy-p-(1-propenyl)-phenol and p-ethyl-m-methoxyphenol. However, the thermo-catalytic reforming of pyrolysis vapors at 500 °C was more efficient for deoxygenation, favoring the formation of benzene, toluene, xylene and ethylbenzene, since the increase in temperature modifies the structure of the catalyst for selectivity of these compounds.

The energetic use of this biomass through catalytic flash pyrolysis can be an alternative to value agricultural by-products with the generation of an extract rich in monoaromatics and polyaromatics that serve as base for resins, dyes and solvents, in addition to the production of charcoal that can be used as a soil enricher improving its moisture holding capacity and increasing nutrient retention, with zero emission. Aromatics are a chemical base for a variety of products in the industry, which currently come almost entirely from the oil industry.
